# The Engineering Side of Hospital Work

**Published:** 1905-04-01

**Authors:** 


### THE ENGINEERING SIDE OF HOSPITAL WORK.

*Continued from page 470.*

### VIII.—LAUNDRIES.

The laundry, it goes without saying, whether it is part of the hospital, or of some other establishment, is a very important part of the hospital arrangements. In medicine, and in surgery, above all things, it is admitted that cleanliness is of the very first importance. And it is not only the question of the sanitary value, and of the healing value of cleanliness, but, as in all hospital matters, there is the sentimental side. The giver is ever so much more cheerful when the arrangements of the hospital meet with his approval, and while he can usually know little as to the medical or surgical efficiency of the arrangements, he can always and does always form very decided opinions as to the matter of cleanliness. But cleanliness, like everything else in this world that is good, costs money. To preserve the spotlessness of everyone and everything an enormous quantity of laundry work has to be done. If hospitals were dependent upon the old methods, the strength and ability to rub, and to wring, of washerwomen such an army of them would be required that there would be very little room for anyone else. Hence machinery has been called to the aid of hospital administration in this as in so many other matters.

Laundry machinery, though it performs an office that we are accustomed to look upon as menial, is among some of the most beautiful of engineering appliances. It is perhaps hardly appreciated that there is great beauty in a great deal of machinery, though it may be doing work that is not at all beautiful. The way in which the forces of nature are called to assist man in some of his difficult and dirtiest work is often very beautiful, and laundry machinery takes high rank in this matter.

Washing clothes is a chemico-physical operation. The dirt has to be removed from the pores, and the surfaces of the clothes, partly by dissolving the dirt in water, or in water containing solutions of soap and soda, and partly by mechanically disturbing the particles of dirt so that the water and solutions can get to them.

The operation of washing consists of four parts: removing the dirt from the clothes, clearing the soap and soda solutions out of the pores of the clothes, removing the water from the clothes, and the process known as finishing. And for the purpose there are washing machines which perform the work previously done by the arms, the board, or the dolly-machines, which remove the greater part of the water from the clothes, taking the place of the old time process of wringing; drying apparatus, which remove the remainder of the moisture, taking the place of the line on which clothes are hung out of doors; and ironing and similar machines to perform the work done with the domestic flat iron. There are also several auxiliary machines, such as the apparatus for mixing the soap and soda, the apparatus for starching, blueing, boiling, etc.

### Washing Machines.

By far the greater number of washing machines consist of an iron or wooden cylinder having an inner cylinder of brass, copper, gunmetal, or wood. The outer cylinder is stationary, and is fitted with apparatus for the delivery of water and steam, also with steam and water gauges, the latter to enable the height of the water inside the cylinder to be seen. The inner cylinder is made sometimes in the form of a cage, as in one of Messrs. Manlove, Alliott's machines, sometimes in the form of a drum with numerous perforations, designed to allow the water, soap solution, etc., to pass through freely. In one case also, that made by Messrs. W. F. Baker and Co., the inner cylinder has projections on each end, forming a fan, and spiral projections round the outside of the body. The inside of the inner cylinder usually carries what is termed a midfeather, a projection of wood, at one or both ends of a diameter of the cylinder, the object being to rub the clothes as they pass. In some forms, also, as in Messrs. Bradford's, there are other smaller wooden projections on the inside of the cylinder arranged for the same purpose. And again, as in Messrs. Bradford's, the inner cylinder, or cage as it is sometimes called, has its longitudinal surface corrugated. The inner cylinder is sometimes divided, by carrying the midfeather right across, into two compartments, or again into three, the rubbing pieces being multiplied in each compartment. The inner cylinder is carried on an axle, which runs in bearings in the ends of the outer cylinder, and is given a rotary motion, the direction of rotation being reversed every few revolutions by automatic machinery attached to the driving gear. The object of reversing the rotation is to prevent the clothes forming themselves into a rope, as they are apt to do if the motion is always in one direction. In addition to the rotary motion given to the inner cylinder, it is also given a second motion of some kind. In some forms the cage is given a longitudinal motion; at certain intervals in others, as in the machines of Messrs. Bradford, the cage has an eccentric motion, causing it to rise partly out of the water, and to be pushed down into it at a period of the revolution. In working the machine, the cage is partly filled with clothes, its door closed and that of the outer cylinder; then water is run in till the apparatus is about half full, and steam is allowed to pass into the water till the temperature is from 80° to 90° Fahr., and the machine rotated. The objects of the perforation, the cage form, and the various motions that have been described as given to the cage are to produce a continual circulation of the water with its contents all through the clothes. For successful washing, every interstice must be explored by the soap solution. Usually, clean water is run in first to clear away any loose dirt that may be present, then water and soapy liquor at the temperature named, and the machine worked for from 20 to 40 minutes according to the kind of clothes that are being washed; then clean water run through again to clear all the dirt-laden solution off, then water is run in again, and steam sufficient to boil the water, the boiling going on for from 20 to 40 minutes according to the substance of the clothes; then the clothes are rinsed three times with clean cold water, and after the last operation, when all the dirt and all the dirt-laden solution is supposed to have been driven out, the clothes are passed on to the apparatus arranged to perform the operation of wringing before going to the drying-room or closet.

(*To be continued.*)

